# Bacteriological quality evaluation of seawater and oysters from the Hansan-Geojeman area in Korea, 2011–2013: impact of inland pollution sources

**DOI:** 10.1186/s40064-016-3049-9

**Published:** 2016-08-24

**Authors:** Jong Soo Mok, Tae Seek Lee, Poong Ho Kim, Hee Jung Lee, Kwang Soo Ha, Kil Bo Shim, Ka Jeong Lee, Yeoun Joong Jung, Ji Hoe Kim

**Affiliations:** 1 Southeast Sea Fisheries Research Institute, National Institute of Fisheries Science, 397-68, Sanyangilju-ro, Sanyang-eup, Tongyeong, 53085 Republic of Korea; 2 Food Safety Research Division, National Institute of Fisheries Science, 216, Gijanghaean-ro, Gijang-eup, Gijang-gun, Busan, 46083 Republic of Korea

**Keywords:** Water quality, Hansan-Geojeman area, Oyster, Fecal bacteria, Wastewater, Pollution source

## Abstract

From 2011 to 2013, we conducted a full sanitary survey of pollution sources in proximity to a shellfish growing area in the Hansan-Geojeman region in Korea, which includes a designated shellfish growing area. In the sea area, 1152 seawater and 209 oyster samples were collected and examined to evaluate their bacteriological quality. There were 758 potential pollution sources in the drainage area, including 40 sources discharging water in 2013. Fecal coliform (FC) concentrations and impact radii of discharges ranged from 1.8 to 700,000 MPN/100 mL and from 3 to 600 m, respectively; however, the pollutants did not reach the designated area. This demonstrates that the dilution of waste was sufficient such that no significant impact occurred within the designated shellfish growing area. The variation in the FC levels of seawater was closely related to season and rainfall. The FC levels of seawater and oysters from the designated area met the regulation limits set by various countries. No pathogens were found in any oysters. The results of the survey indicate that the oysters produced in this area are apparently safe for raw consumption based on their bacterial quality.

## Background

Shellfish are an important global food resource, but human and animal fecal pollution of inland and coastal waters may negatively impact shellfish sanitary status (Feldhusen 2000; Dorfman and Sinclair Rosselot 2008) resulting in economic losses due to shellfish bed closures (Rabinovici et al. 2004). For many decades, consumption of raw or undercooked shellfish such as oysters has been implicated in numerous food poisoning outbreaks due to pathogenic microorganisms (Rippey 1994; Potasman et al. 2002; Iwamoto et al. 2010).

Fecal pollution can also deteriorate the aquatic environment for producing, harvesting, and consuming shellfish. To control and improve the water quality, proper management and remediation plans require good methods. Fecal coliforms (FCs) including *Escherichia coli* (*E*. *coli*) are used as indicators of the quality of shellfish and to classify the growing areas from which shellfish are harvested (Hunt 1977). Fecal contamination can be hazardous to humans, and therefore the levels of FCs in bivalves or their growing areas must be monitored regularly to determine whether shellfish are safe for consumption. To protect public health, the authorities in various countries, such as Korea, the United States (US), the European Union (EU), and New Zealand, have established regulatory limits and monitoring programs using the FC levels of bivalves or their growing areas (European Commission (EC) 2005; New Zealand Food Safety Authority (NZFSA) 2006; US Food and Drug Administration (FDA) 2013; Korea Ministry of Food and Drug Safety (KMFDS) 2015; Ministry of Oceans and Fisheries (MOF) 2015). The impact of inland pollution sources on the bacteriological water quality of shellfish growing areas must also be estimated regularly to identify fecal pollution sources and evaluate the growing areas.

The Food and Agriculture Organization (FAO) of the United Nations (FAO 2013) reported that Korea was the world’s third largest producer of mollusks in 2013, with 293,773 tons of production, accounting for almost 2.0 % of global production. According to Statistics Korea (2013), the country produced 239,779 tons of oysters in 2013, the largest amount of shellfish produced in Korea. In particular, Gyeongnam province, located in the south of Korea, produced the largest amount of oysters in Korea, accounting for ~90 % of oyster products. The products are consumed domestically or exported mainly to the US, Japan, and the EU (Mok et al. 2013, 2014a, 2015). The Korean government has established a memorandum of understanding with the US and EU, and there are designated shellfish growing areas for export along the southern coast of Korea which meet the standards set by these countries (Mok et al. 2014a, 2015). The Hansan-Geojeman area, located in Gyeongnam province, has been designated a shellfish growing area for ex-port because it is a major oyster production area in Korea (Ha et al. 2011). Oysters are commonly consumed raw in many cultures, including Korea. Therefore, the sanitary status of seawater in this area must be known to assess oyster quality both for Korean populations and consumers in importing countries.

In this study, we determined the concentrations of total coliforms (TCs) and FCs in seawater samples collected from the Hansan-Geojeman area, including a designated shellfish growing area for export, on the southern coast of Korea, and evaluated the bacteriological quality of the area. The density and bioaccumulation of FCs in the oysters were determined at the same sites as the seawater samples were collected. In addition, we also attempted to evaluate the impacts of inland pollution sources on the bacteriological water quality of the area. To our knowledge, this is the first report on a full sanitary survey for all inland pollution sources, seawater, and oysters collected from the Hansan-Geojeman area in Korea.

## Methods

### Sample collection

**Fig. 1:**
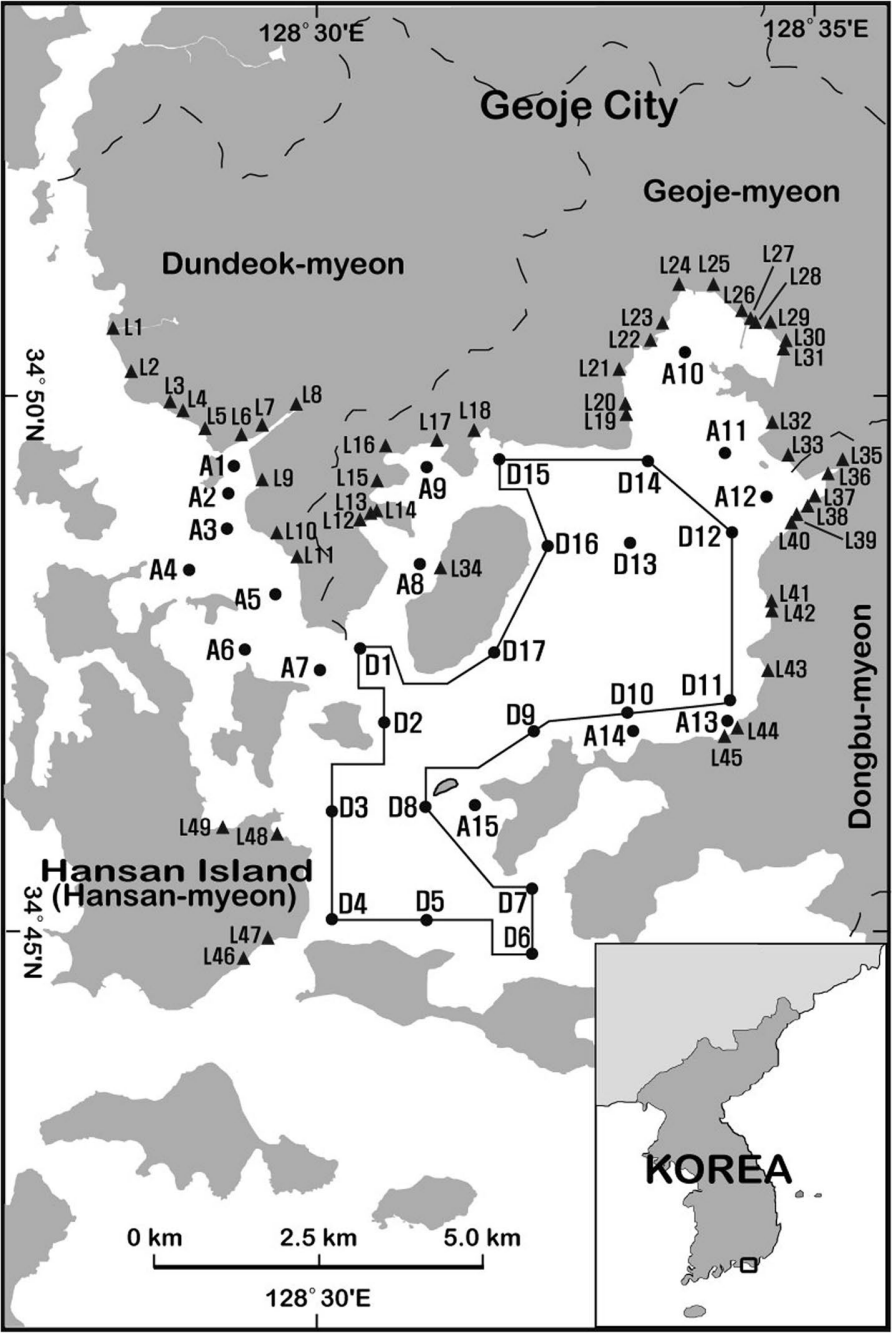
Sampling locations of inland pollution sources (*black filled triangle*), seawater (*black filled circle*), and oysters from the Hansan-Geojeman area on the southern coast of Korea. A *black closed line* indicates the boundary line of the designated shellfish growing area

The seawater sampling stations were located based on various potential fecal pollution sources and geographical conditions in the Hansan-Geojeman area on the southern coast of Korea, where a designated shellfish growing area for export is located (Fig. 1). To evaluate the bacteriological water quality of the shellfish growing area based on criteria set by the Korean Shellfish Sanitation Program (KSSP; MOF 2015) and the National Shellfish Sanitation Program (NSSP; US FDA 2013), the seawater sampling should be undertaken over a 3-year period. The seawater samples were collected once a month from 2011 to 2013 at the 38 monitoring stations in the area. In this area, 612 seawater samples were collected from 17 stations (D1–D17) located in the designated shellfish growing area, while 540 samples were collected from 15 stations (A1–A15) in the adjacent area. The seawater samples were collected at a depth of 10 cm below the surface into sterilized wide-mouth bottles (250 mL) and stored in a stainless steel container.

Oyster samples (*Crassostrea gigas*) were collected at six monitoring stations during the same periods as the seawater samples (Fig. 1). In the designated area, 141 oyster samples were collected from stations D2, D5, D9 and D14, while in the adjacent area, 68 samples were collected from stations A3 and A8. The oyster samples were collected at depths of 2–3 m from a hanging rope culture, and stored in Whirl–Pak bags (25.4 × 50.8 cm, Nasco).

A sanitary survey requires the identification and evaluation of all environmental factors, including actual and potential pollution sources that have a bearing on the water quality in the shellfish growing area. When the on-site shoreline surveys for inland pollution sources in the drainage area of the Hansan-Geojeman area were performed in March 2009 and October 2013, there were 758 direct and indirect pollution sources in the drainage area. Among them, 49 sources, classified as actual pollution sources, discharged water and were chosen as sampling station locations to evaluate the impacts of inland pollution sources on the bacteriological water quality of the shellfish growing area (Fig. 1). The samples were collected into sterilized wide-mouth bottles (250 mL) and stored in a stainless steel container.

All samples for microbiological analysis were maintained below 10 °C during transport to the laboratory. Water temperature and salinity were measured at the depths at which the seawater samples were collected using a YSI 556 Multiprobe System (YSI, Yellow Springs, OH, USA).

### Fecal pollution-indicative bacteria analysis

The bacteriological examination of the water samples was performed within 6 h after collection. The oyster samples were immediately washed with tap water after returning to the laboratory and shucked for bacteriological examination. Each oyster sample was composed of more than 12 animals collected from the same station. The TCs and FCs in the samples were examined according to the recommended procedures for the examination of seawater and shellfish (American Public Health Association (APHA) 1970). TC and FC counts were determined by a five-tube decimal dilution test using the most probable number (MPN) method. Lauryl tryptose broth (Difco, Detroit, MI, USA) was used as the presumptive medium. The presumptive positive culture tubes, in which gas formed within 48 h after inoculation at 35.0 ± 0.5 °C, were confirmed for TCs in brilliant green lactose bile broth (Difco), cultured for 24–48 h at 35.0 ± 0.5 °C. The FCs were confirmed in EC medium (Difco), cultured for 24 h at 44.5 °C. TC and FC populations were expressed as MPN per 100 mL or 100 g.

### Evaluation of inland pollution sources

The flow velocities of discharges from inland pollution sources were measured on-site using a hydrometer (Flo-Mate 2000, Marsh McBirney, Loveland, CO, USA), and flow rates were calculated using the velocity-area method. This is the pollution source evaluation method suggested by the US FDA in accordance with the equations presented below (Shim et al. 2012). Thus, it is calculated as the amount of dilution water required to dilute the FC density to less than the standard level of 14 MPN/100 mL in the seawater samples based on the US FDA guidelines (US FDA 2013). The method assumes that the discharges from pollution sources are distributed equally into receiving water without water column.

$${\text{Daily load}}\,{\text{(DL)}}\, = \,{\text{FC concentration}}\, \times \,{\text{Flow}},$$

where DL is expressed as MPN/day, FC concentration is expressed as MPN/100 mL, and Flow is L/min.

$${\text{Dilution water required}}\,{\text{(DWR)}}\, = \,{\text{DL}}/{\text{standard level}},$$

where DWR is m^3^/day and Standard level is 14 MPN/100 mL.

$${\text{Dilution water required}}\,{\text{(DWR)}}\, = \,{\text{DL}}/{\text{standard level}},$$

where IA is expressed as m^2^/day and AD is the average depth expressed in meters.

$${\text{Radius of half-circle}}\,{\text{(CHR)}}\, = \,{\text{Square root}}({\text{IA}} \times {2/3.14)},$$

where CHR is expressed as m/day.

### Evaluation of seawater quality

The sanitary conditions of seawater in the designated and adjacent areas of the Hansan-Geojeman area were evaluated according to the sanitation standard operating procedure for a designated area for shellfish production suggested by the KSSP (MOF 2015) and the NSSP (US FDA 2013). Thus, the sanitary condition of each station was evaluated based on the geometric mean and estimated 90th percentile (Est. 90th) of TCs and FCs in samples collected more than 30 times over 3 years. The estimated 90th percentile was calculated as follows:

$${\text{Est. 90th}}\,=\,{\text{Antilog ([Slog] 1.28}}\,+\,{\text{Xlog}}),$$

where Slog = the standard deviation of the log value of each MPN in each data group and Xlog = the average log value of each MPN in each data group.

### Statistical analysis

Statistical evaluation was conducted using analysis of variance with the general linear model procedure (SAS version 9.2, SAS Institute, Cary, NC, USA). Duncan’s multiple-range test was applied to determine the significance of differences between the numbers of coliform bacteria.

## Results and discussion

### FC concentrations and evaluation of inland pollution sources

**Fig. 2:**
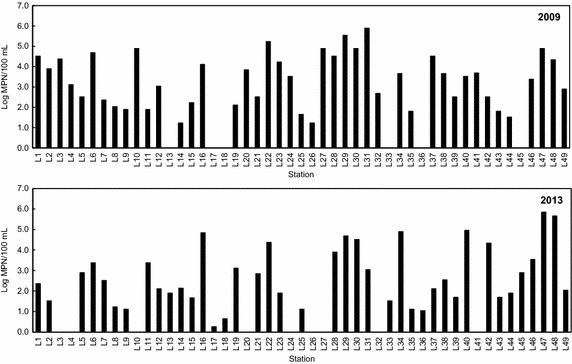
Spatial distribution of fecal coliform concentrations of inland pollution sources in the drainage area of the Hansan-Geojeman area, Korea

The FC concentrations in the inland pollution sources collected from 49 stations in the drainage area of the Hansan-Geojeman area are shown in Fig. 2 and Tables 1 and 2. These results revealed that the main pollution sources of the drainage area were stream water, domestic wastewater, land-based fish farm wastewater, and small food processing plant wastewater. No industrial wastewater or livestock wastewater was involved. The drainage area consists of four small regions including Dundeok-myeon, Geoje-myeon, and Dongbu-myeon in Geoje City, and Hansan-myeon in Tongyeong City, according to the locations of shoreline pollution sources. Approximately 17,550 people lived in this area (161.8 km^2^) in 2012. Of the total land area, 72.3 % (116.9 km^2^) was occupied by forestry fields, and 18.5 % was cultivated as both rice paddy and dry paddy (Statistics Korea 2013).

**Table 1:** The evaluation of discharges from inland contamination sources of Dundeok-myeon and Geoje-myeon in the drainage area of the Hansan-Geojeman area, Korea

Station	TD	DV (L/min)	FC (MPN/100 mL)	AD (m)	DL (MPN/day)	DWR (m^3^)	IA (m^2^)	RHC (m)
Dundeok-myeon								
L1	SW	154	230	1	5.1 × 10^8^	3.6 × 10^3^	3.6 × 10^3^	48
L2	SW	20	33	1	9.5 × 10^6^	6.8 × 10^1^	6.8 × 10^1^	7
L5	SW	115	790	1	1.3 × 10^9^	9.3 × 10^3^	9.3 × 10^3^	77
L6	SW	54	2400	1	1.9 × 10^9^	1.3 × 10^4^	1.3 × 10^4^	92
L7	SW	124	330	1	5.9 × 10^8^	4.2 × 10^3^	4.2 × 10^3^	52
L8	SW	4000	17	1	9.8 × 10^8^	7.0 × 10^3^	7.0 × 10^3^	67
L9	SW	10	13	1	1.9 × 10^6^	1.3 × 10^1^	1.3 × 10^1^	3
L11	SFPW	240	2400	1	8.3 × 10^9^	5.9 × 10^4^	5.9 × 10^4^	194
Geoje-myeon								
L12	DW	25	130	1	4.7 × 10^7^	3.3 × 10^2^	3.3 × 10^2^	15
L13	SW	15	79	1	1.7 × 10^7^	1.2 × 10^2^	1.2 × 10^2^	9
L14	SW	12	140	1	2.4 × 10^7^	1.7 × 10^2^	1.7 × 10^2^	10
L15	SW	40	46	1	2.6 × 10^7^	1.9 × 10^2^	1.9 × 10^2^	11
L16	SW	18	70,000	2	1.8 × 10^10^	1.3 × 10^5^	6.5 × 10^4^	203
L17	LFFW	2000	1.8	1	5.2 × 10^7^	3.7 × 10^2^	3.7 × 10^2^	15
L18	SW	55	4.5	1	3.6 × 10^6^	2.5 × 10^1^	2.5 × 10^1^	4
L19	SW	10	1300	1	1.9 × 10^8^	1.3 × 10^3^	1.3 × 10^3^	29
L21	SW	380	700	2	3.8 × 10^9^	2.7 × 10^4^	1.4 × 10^4^	93
L22	SW	56	24,000	2	1.9 × 10^10^	1.4 × 10^5^	6.9 × 10^4^	210
L23	SW	100	79	1	1.1 × 10^8^	8.1 × 10^2^	8.1 × 10^2^	23
L25	SW	2500	13	1	4.7 × 10^8^	3.3 × 10^3^	3.3 × 10^3^	46
L28	SW	1000	7900	3	1.1 × 10^11^	8.1 × 10^5^	2.7 × 10^5^	415
L29	DW	100	49,000	3	7.1 × 10^10^	5.0 × 10^5^	1.7 × 10^5^	327
L30	SW	500	33,000	3	2.4 × 10^11^	1.7 × 10^6^	5.7 × 10^5^	600
L31	SW	440	1100	1	7.0 × 10^9^	5.0 × 10^4^	5.0 × 10^4^	178
L33	SW	4000	33	1	1.9 × 10^9^	1.4 × 10^4^	1.4 × 10^4^	93
L34	DW	10	79,000	2	1.1 × 10^10^	8.1 × 10^4^	4.1 × 10^4^	161

*TD* type of discharge; *DV* discharge volume; *FC* fecal coliform; *AD* average depth; *DL* daily load; *DWR* dilution water required; *IA* impact area; *RHC* radius of half-circle; *SW* stream water; *DW* domestic wastewater; *SFPW* small food processing plant wastewater; *LFFW* land-based fish farm wastewater

**Table 2:** The evaluation of discharges from inland contamination sources of Dongbu-myeon and Hansan-myeon in the drainage area of the Hansan-Geojeman area, Korea

Station	TD	DV (L/min)	FC (MPN/100 mL)	AD (m)	DL (MPN/day)	DWR (m^3^)	IA (m^2^)	RHC (m)
Dongbu-myeon								
L35	SW	5300	13	1	9.9 × 10^8^	7.1 × 10^3^	7.1 × 10^3^	67
L36	SW	330	11	1	5.2 × 10^7^	3.7 × 10^2^	3.7 × 10^2^	15
L37	SW	54	130	1	1.0 × 10^8^	7.2 × 10^2^	7.2 × 10^2^	21
L38	SW	16	350	1	8.1 × 10^7^	5.8 × 10^2^	5.8 × 10^2^	19
L39	SW	28	49	1	2.0 × 10^7^	1.4 × 10^2^	1.4 × 10^2^	9
L40	DW	15	92,000	2	2.0 × 10^10^	1.4 × 10^5^	7.1 × 10^4^	213
L42	DW	10	22,000	1	3.2 × 10^9^	2.3 × 10^4^	2.3 × 10^4^	120
L43	SW	24	49	1	1.7 × 10^7^	1.2 × 10^2^	1.2 × 10^2^	9
L44	SW	120	79	1	1.4 × 10^8^	9.8 × 10^2^	9.8 × 10^2^	25
L45	SW	42	790	1	4.8 × 10^8^	3.4 × 10^3^	3.4 × 10^3^	47
Hansan-myeon								
L46	SW	30	3500	1	1.5 × 10^9^	1.1 × 10^4^	1.1 × 10^4^	83
L47	DW	50	700,000	7	5.0 × 10^11^	3.6 × 10^6^	5.1 × 10^5^	572
L48	DW	70	460,000	7	4.6 × 10^11^	3.3 × 10^6^	4.7 × 10^5^	549
L49	SW	100	110	1	1.6 × 10^8^	1.1 × 10^3^	1.1 × 10^3^	27

*TD* type of discharge; *DV* discharge volume; *FC* fecal coliform; *AD* average depth; *DL* daily load; *DWR* dilution water required; *IA* impact area; *RHC* radius of half-circle; *SW* stream water; *DW* domestic wastewater

In the inland area, FC concentrations were measured at 43 stations in 2009 and at 40 stations in 2013 (Fig. 2). The other inland pollution sources did not discharge during the sampling period. Surface water impairment due to point source (PS) and nonpoint source (NPS) pollution threatens aquatic ecosystems and water quality. PS pollution mainly includes municipal sewage discharges (from urban or densely populated areas) and industrial wastewater loads (from a variety of manufacturers). NPS pollution occurs when rainfall, snowmelt water, or irrigation water flows over land, carrying and depositing pollutants into streams, lakes, and coastal waters (Wu and Chen 2013). Detection of bacterial and viral indicators by culture or molecular techniques may be influenced by rainfall events, and therefore rainfall is often not considered when monitoring the microbial quality of waters (Lipp et al. 2001; Rose et al. 2001). Therefore, this shoreline survey was conducted during dry periods within the harvest season of oysters from October to May of the following year to minimize NPS inputs to the inland pollution sources, as no precipitation occurred. In 2009, the FC concentrations of pollution sources ranged from 17 to 790,000 MPN/100 mL (1.2–5.9 log MPN/100 mL); the highest level was found at station L31, the Namdong Stream in Geoje-myeon. The FC concentrations of pollution sources in 2013 ranged from 1.8 to 700,000 MPN/100 mL (0.3–5.8 log MPN/100 mL); the highest level was found at station L47, domestic wastewater from Jindu Village in Hansan-myeon. In 2009, the total flow rate and FC daily load from 43 stations were 17,875 L/min and 2.3 × 10^12^ MPN/day, respectively (data not shown). In 2013, the total flow rate and FC daily load from 40 stations were 22,167 L/min and 1.5 × 10^12^ MPN/day, respectively (Tables 1, 2). Therefore, the daily load of FCs from the discharges in the drainage area of the Hansan-Geojeman area did not differ significantly between 2009 and 2013.

When the on-site shoreline survey for inland pollution sources in the drainage area of the Hansan-Geojeman area was performed in 2013, there were 758 potential pollution sources in the area. Among them, 40 sources had discharged water as actual contamination sources, including eight sources in Dundeok-myeon, 18 sources in Geoje-myeon, 10 sources in Dongbu-myeon, and four sources in Hansan-myeon (Fig. 1; Tables 1, 2). The diffusion range of discharged water from inland pollution sources in 2013 was estimated according to the formula in the “Methods” section, and the results are summarized in Tables 1 and 2. For the 40 inland water samples, the total flow rate was 22,167 L/min with a range of 10–5300 L/min. The discharges were composed of 19,647 L/min of stream water, 280 L/min of domestic wastewater, 240 L/min of small food processing plant wastewater, and 2000 L/min of land-based fish farm wastewater. The FC concentrations of discharges ranged between 1.8 and 700,000 MPN/100 mL. The FC daily load ranged from 1.9 × 10^6^ to 5.0 × 10^11^ MPN/day. According to the formula, the affected radii of discharges in this sea area ranged from 3 to 600 m, among which radii greater than 500 m were found at stations L30, L47 and L48; however, the pollutants did not reach the boundary line of the designated area because of a buffer zone between the shoreline and boundary line.

The flow rates of two domestic wastewaters (L47 and L48) from Jindu Village in Hansan-myeon were relatively low, in the range of 50–70 L/min, however, their FC levels were very high, in the range of 460,000–700,000 MPN/100 mL. The radii of calculated impact areas of these sources ranged from 549 to 572 m. The domestic wastewaters were very large contamination sources in the drainage areas around the Hansan-Geojeman area. The daily loads and impact radii of pollution sources for FCs in Geoje-myeon were relatively large compared to other areas. In particular, the sources (L19–L31) of the sub-area in Geoje-myeon located in the north part of the Hansan-Geojeman area were the major contributors of wastewater pollution to the area (Fig. 1). The total discharge rate of pollution sources in the sub-area, including nine stream waters and one domestic wastewater, was 5086 L/min. The FC concentrations and impact radii of pollution sources ranged from 13 to 49,000 MPN/100 mL and from 23 to 600 m, respectively, of which three sources (L28, L29 and L30) had affected radii >300 m due to a relatively densely populated residential area in the drainage area. The highest values for the FC daily load and impact radius among discharges around the Hansan-Geojeman area were found at site L30, the Seojeong Stream in Geoje-myeon (Table 1).

According to these results, along with the large affected radii of L28, L29 and L30 in Geoje-myeon, and L47 and L48 in Hansan-myeon, these sources are clearly identified as significant pollution sources. Therefore, based on the recommendations given by our official paper (Yoo et al. 2014), the local government authorities in Korea, such as in Tongyeong City and Geoje City, will construct a new wastewater treatment plant (WWTP) or enlarge a currently operating WWTP in these regions to protect the Hansan-Geojeman area, including the designated shellfish growing area. This implies that the inland waters in Geoje-myeon are highly polluted by various pollutants from stream water and domestic wastewater throughout the populated residential area. However, the pollutants do not reach the boundary line of the designated area due to the existing buffer zone.

### Bacteriological water quality

**Fig. 3:**
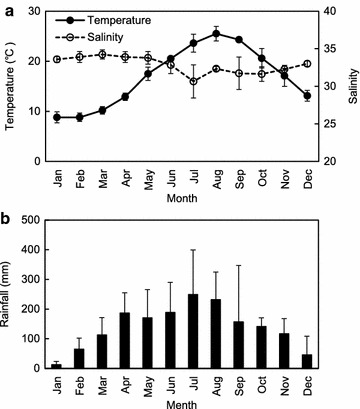
Monthly variations in mean water temperature (**a**), salinity (**a**), and rainfall (**b**), in the Hansan-Geojeman area, Korea from 2011 to 2013. The *scale bars* represent standard deviations. Rainfall data were obtained from the meteorological observatory of Geoje City

**Fig. 4:**
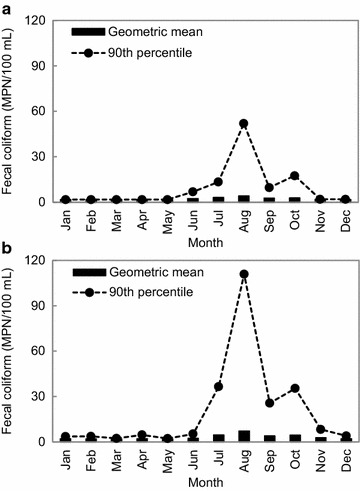
Monthly variation of the geometric mean and estimated 90th percentile of fecal coliform levels for seawater samples collected in the designated shellfish growing area (**a**) and adjacent area (**b**) of the Hansan-Geojeman area, Korea from 2011 to 2013

**Fig. 5:**
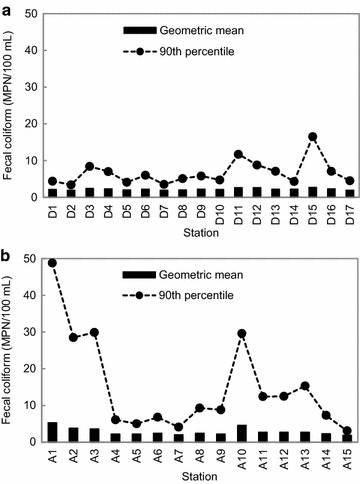
Spatial distribution of the geometric mean and estimated 90th percentile of fecal coliform levels for seawater samples collected in the designated shellfish growing area (**a**) and adjacent area (**b**) of the Hansan-Geojeman area, Korea from 2011 to 2013

Environmental conditions and FC levels at seawater sampling stations in the Hansan-Geojeman area from 2011 to 2013 are summarized in Figs. 3, 4 and 5. Monthly mean variations in the surface water temperature and salinity at all stations throughout the monitoring period are shown in Fig. 3a. During the survey period, the monthly mean water temperature ranged from 8.8 ± 0.8 °C in February to 25.5 ± 1.4 °C in August. The monthly mean surface salinity varied from 30.65 ± 2.20 to 34.23 ± 0.31 practical salinity units (psu) with seasonal variation. The lowest salinity was recorded in July due to heavy rainfall during the wet season. However, the differences in water temperature and salinity among sampling stations were not significant. Monthly mean variations in rainfall during the 3 years are shown in Fig. 3b. Monthly mean rainfall was relatively high between July and August at 248.8 ± 150.3 and 231.2 ± 93.5 mm, respectively.

In addition, monthly FC variations at seawater stations in the Hansan-Geojeman area from 2011 to 2013 are shown in Fig. 4. In the designated area, the monthly values of the FC geometric mean and estimated 90th percentile ranged from 1.8 to 4.2 and 1.8 to 52.0 MPN/100 mL, respectively; the highest values were found in August (Fig. 4a). In the adjacent area, the monthly values of the FC geometric mean and estimated 90th percentile ranged from 1.8 to 7.1 and 2.2 to 110.9 MPN/100 mL, respectively, the maximum levels were also found in August (Fig. 4b). The bacteriological water quality was very favorable in the designated area, with the exception of samples collected after rainfall (52 mm) in August 2011. Seasonal temperature variation may affect the abundance of bacteria (Rippey 1994). Bacteria such as coliforms may proliferate in nutrient-rich waters during warmer months and survive in colder months (Anderson et al. 1983). Avila et al. (1989) described the monitoring of seawater quality on the southern coast of Spain and reported values of microbial counts twice as high in fall and spring and around eight-fold higher in summer than in winter. Chigbu et al. (2004) also reported that FC geometric mean levels exhibited a positive relationship with rainfall in the Mississippi Sound. This study also revealed a similar result, with relatively higher FC counts detected in the summer season than in other seasons which were affected greatly by rainfall. Therefore, the results indicate that FC concentrations had seasonal variation, and in particular, were the highest after the heavy rainfall before sampling in August 2011. However, because the oyster harvesting period in Korea was from October to May of the following year, oysters were, fortunately, not harvested during the summer season with heavy rainfall.

The values of the FC geometric mean and estimated 90th percentiles at seawater sampling sites in the Hansan-Geojeman area from 2011 to 2013 are summarized in Fig. 5. In the designated area, the values of the FC geometric mean and estimated 90th percentile at each station ranged from 1.9 to 2.7 MPN/100 mL and from 3.4 to 16.5 MPN/100 mL, respectively; the highest values were found at site D15 (Fig. 5a). In the adjacent area, the values of the FC geometric mean and estimated 90th percentile at each station ranged from 1.9 to 5.3 MPN/100 mL and from 3.1 to 48.8 MPN/100 mL, respectively; the maximums were found at site A1 (Fig. 5b). We assume that the very high FC concentration (1300 MPN/100 mL) in a sample collected at site A1 after rainfall in August 2011 (Fig. 3b) was due to waste discharges from sites L6, L7 and L8 along the shoreline within a populated residential area (Fig. 1).

Seawater stations A1, A2, A3, A5 and D1, which were approximately 0.5, 1.0, 1.5, 2.5 and 4.0 km away, respectively, from the Dundeok Stream mouth in Geoje City (Fig. 1), were established to evaluate the bacteriological water quality of discharged water from the stream. The estimated FC 90th percentiles at each station over 3 years were 48.8, 28.5, 29.9, 5.0 and 4.4 MPN/100 mL, respectively (Fig. 5). On the other hand, FC values and flow rates of discharges from pollution sources (stations L6, L7 and L8) occurring within the Dundeok Stream ranged from 17 to 2400 MPN/100 mL and from 54 to 4000 L/min, respectively (Table 1). In addition, stations A10, A11 and D14 were located to evaluate the impact of pollution sources in Geoje-myeon in the north part of the Hansan-Geojeman area on the water quality of the shellfish growing area (Fig. 1). Seawater station A10 was the closest to the major contributors (stations from L19 to L31), including the Seojeong Stream (L30), and the most impacted by the wastewater pollutions in the Hansan-Geojeman area (Fig. 1; Table 1), as mentioned above. Stations A11 and D14, which is located on the boundary line of the designated area, were approximately 1.5 and 2.0 km away from station A10, respectively. The estimated FC 90th percentiles at the stations (A10, A11 and D14) over 3 years were 29.6, 12.4 and 4.3 MPN/100 mL, respectively (Fig. 5). These results imply that many pollutants flow into the sea area from the pollution sources, however, they are diluted within the buffer zone between the coastline and the designated area, and the bacteria are also reduced due to dilution, decay, or removal in the sea area. Similarly, the previous studies addressed that the reductions in FC concentrations within coastal water from wastewater outfalls were attributed to dilution or overall removal (Azalea et al. 2010; Mok et al. 2016). Chigbu et al. (2005) reported that FC dynamics in coastal waters depends on the amount of bacterial loading from streams and rivers, water mixing and dispersion in receiving waters, and bacterial losses due to death and sedimentation.

In this study, all stations in the designated area showed values far below the regulation limits of the geometric mean and estimated 90th percentile values (14 and 43 MPN/100 mL, respectively) for FCs set by Korea (MOF 2015), the US (US FDA 2013), and New Zealand (NZFSA 2006) for approved areas. On the other hand, in the adjacent area only one station (A1) exceeded the limit of the 90th percentile for FCs. This indicates that the sanitary condition of the designated area in the Hansan-Geojeman area met the criteria of Korea, the US, and New Zealand, and shellfish, including oysters, produced in the area are suitable for raw consumption.

### Concentration and bioaccumulation of coliform bacteria in oysters

The TC and FC geometric mean values in oysters from the six sampling sites in the Hansan-Geojeman area throughout the monitoring period are shown in Table 3. The TC and FC geometric mean values ranged from 65.6 to 127.1 MPN/100 g and from 23.7 to 26.7 MPN/100 g, respectively; the highest value was found in oyster samples collected at site A3 in the adjacent area. FC concentrations in oyster samples ranged from <18 to 230 MPN/100 g, which met the regulation limit of <230 MPN/100 g for *E. coli* (one FC species) set by Korea (KMFDS 2015), New Zealand (NZFSA 2006), and the EU (EC 2005). No pathogens such as *Salmonella* spp. or *Shigella* spp. were found in any oyster samples collected from the Hansan-Geojeman area (data not shown). We also previously reported that the concentrations of harmful metals in all oyster samples from the same area were within the regulatory limits set by Korea and other countries (Mok et al. 2015). The results indicate that the oysters produced in the area do not present an appreciable hazard to human health based on fecal pollution or harmful metals, and are apparently safe for raw consumption.

**Table 3:** The geometric mean value of coliforms in seawaters and oysters, and bioaccumulation factor of fecal coliform in oysters

Station	Seawater			Oyster			BF^a^
	GM (MPN/100 mL)		No. of samples	GM (MPN/100 g)		No. of samples	
	TC	FC		TC	FC		
Adjacent area							
A3	4.6	3.6	36	127.1	26.7	32	7.4
A8	2.6	2.4	36	77.4	23.8	36	9.9
Designated area							
D2	2.3	1.9	36	98.5	23.8	36	12.5
D5	2.2	2.0	36	65.6	25.6	33	12.8
D9	2.4	2.2	36	66.9	25.6	36	11.6
D14	2.4	2.2	36	97.0	23.7	36	10.8

*GM* geometric mean; *TC* total coliform; *FC* fecal coliform

^a^The bioaccumulation factor (BF) was calculated as the geometric mean value of FC levels in 100 g of oyster divided by that in 100 mL of seawater

The bioaccumulation factors of FCs in all oyster samples from the surrounding seawater ranged from 7.4- to 12.8-fold; the highest factor was found at station D5 in the designated area (Table 3). The FC bioaccumulation factors in oysters were slightly higher at stations in the designated area than at stations in the adjacent area, which are closer and more exposed to inland pollution sources, because the difference in FC concentrations between the designated area and the adjacent area was relatively larger in the seawater than in the oysters. This study also indicates that the FC levels in oysters were higher in those collected at the seawater stations with high FC concentrations, however, the FC bioaccumulation factors in oysters were near constant, probably, because the FC in shellfish is accumulated with near saturation level. Although bacteria may usually accumulate in bivalves tissue to levels much higher than in the surrounding water, the accumulation rates differ among various filter-feeders, as their ability to accumulate microorganisms varies from a few fold to more than hundreds-fold (Doré and Lees 1995; Burkhardt and Calci 2000). The accumulation of contaminants in marine organisms depends on both their uptake and elimination rates. Contaminants are taken up by the organisms and concentrated at various levels in the body (Sivaperumal et al. 2007; Abdallah 2013). We previously reported that the bioaccumulation factors of heavy metals in oysters (Mok et al. 2015) and mussels (Mok et al. 2014b) collected from the southern coast of Korea ranged from 1413- to 618,958-fold and from 429- to 74,794-fold, respectively. Burkhardt and Calci (2000) also reported that F-specific coliphage was accumulated selectively up to 99-fold in oysters. Our present findings indicate that oysters accumulate FCs at a relatively slower rate than heavy metals and F-specific coliphage. Therefore, the results imply that the animals accumulate different contaminants in the body at different levels compared to the surrounding seawater.

## Conclusions

This study describes the levels of indicator microorganisms present in point and non-point sources of pollution in the proximity of a shellfish growing area and the impact on the shellfish therein. In addition, analysis of fecal pollution-indicative bacteria in seawater and oysters collected from the Hansan-Geojeman area in Korea, including a designated shellfish growing area, was conducted to evaluate the bacteriological quality of the seawater and oysters.

There were 758 potential pollution sources in the drainage area of the Hansan-Geojeman area and among them, 40 sources discharged water as actual contamination sources in 2013. For the 40 inland water samples, the total flow rate was 22,167 L/min with a range of 10–5300 L/min. FC concentrations of discharges ranged between 1.8 and 700,000 MPN/100 mL. The impact radii of discharges on the shellfish growing area ranged from 3 to 600 m, in which the significant pollution sources with impact radii >300 m were clearly identified as two stream waters (stations L28 and L30) and three domestic wastewaters (stations L29, L47, and L48); however, the pollutants did not reach the boundary line of the designated area due to the existing buffer zone in which bacteria were reduced. Therefore, our study demonstrates that many pollution sources have been identified, and based on monitoring of indicator bacteria in shellfish and seawater, these sources had no significant impact on the sanitary quality of the shellfish from a bacterial standpoint. In addition, since inland pollution sources were the most important variable in predicting FC concentrations in the shellfish growing area, a study of the levels and sources of FC bacteria in the drainage area of the Hansan-Geojeman area is required to develop a management plan for reducing FC bacterial pollution.

This study also revealed that FC counts in seawater samples were relatively higher in the summer season than in other seasons, and were largely affected by rainfall. The bioaccumulation factors of FCs in all oyster samples from the surrounding seawater ranged from 7.4- to 12.8-fold. We confirmed that all seawater stations in the designated area from 2011 to 2013 had FC values far below the regulation limits set by Korea, the US, and New Zealand. FC concentrations in oyster samples ranged from <18 to 230 MPN/100 g, which is within the regulation limits for *E. coli* set by Korea, New Zealand, and the EU. Therefore, these results indicate that the sanitary condition of the designated area in the Hansan-Geojeman area met the criteria of various countries, and the oysters produced from the area are suitable for raw consumption.
